# Panitumumab interaction with TAS‐102 leads to combinational anticancer effects via blocking of EGFR‐mediated tumor response to trifluridine

**DOI:** 10.1002/1878-0261.12074

**Published:** 2017-05-30

**Authors:** Yuji Baba, Toshiya Tamura, Yoshihiko Satoh, Masamitsu Gotou, Hiroshi Sawada, Shunsuke Ebara, Kazunori Shibuya, Jumpei Soeda, Kazuhide Nakamura

**Affiliations:** ^1^ Oncology Drug Discovery Unit Pharmaceutical Research Division Takeda Pharmaceutical Company Limited Fujisawa Japan; ^2^ Integrated Technology Research Laboratories Pharmaceutical Research Division Takeda Pharmaceutical Company Limited Fujisawa Japan; ^3^ Medical Affairs Department Takeda Pharmaceutical Company Limited Tokyo Japan

**Keywords:** colorectal cancer, EGFR, panitumumab, phosphorylation, TAS‐102, trifluridine

## Abstract

Panitumumab is a monoclonal antibody developed against the human epidermal growth factor receptor (EGFR). TAS‐102 is a novel chemotherapeutic agent containing trifluridine (FTD) as the active cytotoxic component. Both panitumumab and TAS‐102 have been approved for the treatment of metastatic colorectal cancer. In this study, we revealed the mechanism underlying the anticancer effects of panitumumab/TAS‐102 combination using preclinical models. Panitumumab/FTD cotreatment showed additive antiproliferative effects in LIM1215 and synergistic antiproliferative effects in SW48 colon cancer cells. Consistent with the *in vitro* effects, panitumumab/TAS‐102 combination caused tumor regression in LIM1215 and COL‐01‐JCK colon cancer patient‐derived xenograft models. In LIM1215 cells, FTD induced extracellular signal‐regulated kinase (ERK)/protein kinase B (AKT)/signal transducer and activator of transcription 3 (STAT3) phosphorylation and subsequent serine/threonine phosphorylation of EGFR, while it had no effects on EGFR tyrosine phosphorylation. Panitumumab and the tyrosine kinase inhibitor erlotinib reduced the basal level of EGFR tyrosine phosphorylation and reversed FTD‐induced ERK/AKT/STAT3 and EGFR serine/threonine phosphorylation. These results suggested that FTD in combination with the basal activity of EGFR tyrosine kinase induced downstream prosurvival signaling through ERK/AKT/STAT3 phosphorylation. Collectively, we propose that panitumumab interacts with FTD by targeting EGFR‐mediated adaptive responses, thereby exerting anticancer effects when used in combination with TAS‐102. These preclinical findings provide a compelling rationale for evaluating the combination of anti‐EGFR antibodies with TAS‐102 against metastatic colorectal cancer.

**Abbreviations**
AKT: protein kinase B
BrdU: 5‐bromo‐2′‐deoxyuridine
CRC: colorectal cancer
DMSO: dimethyl sulfoxide
EGFR: epidermal growth factor receptor
ERK: extracellular signal‐regulated kinase
FBS: fetal bovine serum
FTD: trifluridine
FU: fluorouracil
GAPDH: glyceraldehyde 3‐phosphate dehydrogenase
*KRAS*: Kirsten rat sarcoma viral oncogene homolog
LC/MS/MS: liquid chromatography–tandem mass spectrometry
MAPK: mitogen‐activated protein kinase
mCRC: metastatic colorectal cancer
MeCN: acetonitrile
MEK: mitogen‐activated protein kinase kinase
MP: monophosphate
MS: mass spectrometry
PDX: patient‐derived colon tumor xenograft
PI3K: phosphatidylinositol 3‐kinase
RAF: rapidly accelerated fibrosarcoma kinase
RAS: rat sarcoma GTPase
SILAC: stable isotope labeling with amino acids in cell culture
STAT: signal transducer and activator of transcription
TEAB: triethylammonium bicarbonate
TNF: tumor necrosis factor
TPI: tipiracil hydrochloride
TP: triphosphate
TS: thymidylate synthase

## 1 Introduction

Colorectal cancer (CRC) is the third most commonly diagnosed cancer and the fourth leading cause of cancer‐related deaths worldwide (Ferlay *et al*., 2015). Patients with advanced and unresectable CRC can be eligible for multiple lines of treatment. Three major chemotherapeutic agents [5‐fluorouracil (5‐FU), irinotecan, and oxaliplatin], an antivascular endothelial growth factor antibody (bevacizumab), and two antiepidermal growth factor receptor (EGFR) antibodies (cetuximab and panitumumab) have shown well‐documented clinical activity for the treatment of metastatic CRC (mCRC) (Jonker *et al*., 2007; Van Cutsem *et al*., 2007). Randomized trials in first‐line setting combining cetuximab with FOLFILI (irinotecan/5‐FU/leucovorin) or FOLFOX (oxaliplatin/5‐FU/leucovorin) or panitumumab with FOLFOX demonstrated a significant survival benefit compared with that of chemotherapy alone. Although the use of cetuximab and panitumumab is restricted only to mCRC patients with *KRAS* (Kirsten rat sarcoma viral oncogene homolog) and *NRAS* wild‐type genes owing to the well‐established link between *RAS* (rat sarcoma GTPase) mutations and lack of response to antibodies (Karapetis *et al*., 2008; Amado *et al*., 2008; Siena *et al*., 2009), these EGFR‐targeting monoclonal antibodies have expanded the range of treatment options for mCRC (Heinemann *et al*., 2016).

Mechanistically, both panitumumab and cetuximab competitively inhibit endogenous ligand binding, thereby suppressing the subsequent activation of EGFR, a member of the human ERBB family of receptor tyrosine kinases. EGFR tyrosine kinase activation stimulates the key processes in tumor growth and progress via activation of downstream signaling pathways, including RAS/RAF (rapidly accelerated fibrosarcoma) kinase/mitogen‐activated protein kinase (MAPK) kinase (MEK)/extracellular signal‐regulated kinase (ERK), phosphatidylinositol 3‐kinase (PI3K)–protein kinase B (also known as AKT), and signal transducer and activator of transcription (STAT) pathways (Yarden and Sliwkowski, 2001; Scaltriti and Baselga, 2006; Hynes and Lane, 2005). The lack of benefit from EGFR antibodies in mCRC with a *RAS* mutation, where downstream signaling is activated irrespective of EGFR ligand binding, underscores that signaling inhibition is critically important for the anticancer efficacy of EGFR antibodies.

TAS‐102 is a novel, orally administered combination of a nucleoside analog trifluridine (FTD) and thymidine phosphorylase inhibitor tipiracil hydrochloride (TPI), at a molar ratio of 1:0.5 (Salvatore *et al*., 2015; Peters, 2015). FTD is the active cytotoxic component of TAS‐102, while TPI plays a role in preventing the rapid degradation of FTD to its inactive form by thymidine phosphorylase (Fukushima *et al*., 2000). FTD is sequentially phosphorylated; its monophosphate form (FTD‐MP) transiently inhibits thymidylate synthase (TS), and its triphosphate form (FTD‐TP) is incorporated into DNA (Temmink *et al*., 2007a; Reyes and Heidelberger, 1965; Santi and Sakai, 1971; Eckstein *et al*., 1994; Tanaka *et al*., 2014). TS inhibition is a major mechanism of action of classical fluoropyrimidines such as 5‐FU (Van Triest and Peters, 1999). Although TS inhibition by FTD‐MP may partly account for the antitumor effects of FTD (Santi and Sakai, 1971; Temmink *et al*., 2004), the incorporation of FTD‐TP into DNA and the resulting DNA damage appear to be the major mechanism of action of FTD (Tanaka *et al*., 2014; Suzuki *et al*., 2011; Matsuoka *et al*., 2015). Importantly, TAS‐102 exhibits antitumor activity against FU‐resistant cell lines in preclinical xenograft models (Emura *et al*., 2004a; Emura *et al*., 2004b; van der Velden *et al*., 2016). Compared with the placebo, TAS‐102 provided an overall survival benefit of approximately 2 months in a randomized phase III trial that included patients with refractory (or intolerant) mCRC (Mayer *et al*., 2015).

TAS‐102 is also a promising candidate for combination therapy with other agents that serve as a backbone chemotherapy, especially for the treatment of mCRC refractory to initial 5‐FU‐based chemotherapy. The combination of TAS‐102 and anti‐EGFR antibodies is effective preclinically; however, the exact mechanism underlying the combination effects remains to be elucidated (Tsukihara *et al*., 2015). In the present study, we evaluated the anticancer efficacy and molecular mechanism of a combination of TAS‐102 and panitumumab in *in vitro* and *in vivo* colon cancer models.

## 2 Materials and methods

### 2.1 Cells and reagents

The human colon cancer cell lines SW48 and LIM1215 were obtained from Horizon Discovery (Cambridge, UK) and DS Pharma Biomedical (Osaka, Japan), respectively. SW48 cells were cultured in McCoy's 5A medium (Wako, Osaka, Japan) with 10% fetal bovine serum (FBS). LIM1215 cells were cultured in RPMI 1640 medium (Wako) with 10% FBS, 1 μg·mL^−1^ hydrocortisone (Sigma, St. Louis, MO, USA), 0.6 μg·mL^−1^ insulin (Thermo Fisher Scientific, Waltham, MA, USA), and 10 μm 1‐thioglycerol (Wako). Panitumumab was provided by Amgen, Inc. (Thousand Oaks, CA, USA). Cetuximab was purchased from Merck Serono (Darmstadt, Germany). FTD was purchased from Tokyo Chemical Industry (Tokyo, Japan). TPI was purchased from Biochempartner (Wuhan, China). Erlotinib was purchased from Selleck Chemicals LLC (Houston, TX, USA). U0126, LY294002, and SB203520 were purchased from Wako. Trametinib was purchased from Cayman Chemical Company (Ann Arbor, MI, USA). All antibodies used in the study were purchased from Cell Signaling Technology (Danvers, MA, USA), except anti‐glyceraldehyde 3‐phosphate dehydrogenase (GAPDH) antibody (Merck Millipore, Billerica, MA, USA).

### 2.2 Cell proliferation and clonogenic assay

For the cell proliferation assay, colon cancer cells were plated in 96‐well plates at a density of 1 × 10^3^ cells per well. Serial dilutions of FTD, panitumumab, and FTD/panitumumab as well as dimethyl sulfoxide (DMSO; control) were added to the culture media 24 h after cell plating. The cells were then cultured for an additional 72 h, and cell viability was determined by the CellTiter‐Glo assay (Promega, Fitchburg, WI, USA). For the clonogenic assay, 1 × 10^3^ SW48 or LIM1215 cells were plated in each well of six‐well plates and subsequently treated with FTD, panitumumab, FTD/panitumumab in combination, or DMSO for 14 days. The cell colonies were stained with 0.5% crystal violet and counted using a GelCount colony counter (Oxford Optronix, Abingdon, UK) (Franken *et al*., 2006).

### 2.3 Analysis of drug combination effects

Calculation of the combination metrics was performed as described previously (Garcia *et al*., 2014). Briefly, isobologram analysis was used to determine the effects of drug combinations. A nine‐parameter response surface model was used to fit the relationship between normalized viability and drug concentrations (Minto *et al*., 2000). To quantify the combined effects of two drugs, a combination index (CI) (Berenbaum, 1985; Chou and Talalay, 1984) or nonlinear blending (Peterson and Novick, 2007) was computed. A CI value below 0.7 was classified as synergy, while a value above 1.3 was classified as antagonism. A value in the range between 0.7 and 1.3 was considered to be additive. Nonlinear blending was applied to determine synergy if the maximum inhibition by a single agent was less than 50%. A blending value above 20 was classified as synergy, while that below −20 was classified as antagonism.

### 2.4 Western blotting

LIM1215 cells were plated at a density of 5 × 10^5^ cells per well in six‐well plates. One day later, the cells were treated with FTD, panitumumab, erlotinib, U0126, LY294002, SB203520, or DMSO for 24 h. The cells were then washed once with cold phosphate‐buffered saline and lysed in lysis buffer [62.5 mm Tris/HCl (pH 7.5), 10% glycerol, 1% SDS] supplemented with protease inhibitor cocktail set II and phosphatase inhibitor cocktail set III (Merck Millipore). After centrifugation, the protein concentrations of the cell lysates were determined using the bicinchoninic acid (BCA) protein assay reagent (Thermo Fisher Scientific). The cell lysates were mixed with Laemmli SDS sample buffer, heated, and subjected to SDS/PAGE, followed by immunoblotting. Detection was performed using an enhanced chemiluminescence reagent (GE Healthcare, Chicago, IL, USA).

### 2.5 Stable isotope labeling with amino acids in a cell culture‐based phosphoproteomics analysis

LIM1215 cells were cultured in stable isotope labeling with amino acids in cell culture (SILAC) K8R10 medium for heavy samples or K0R0 medium for light samples (Thermo Scientific), supplemented with 10% dialyzed FBS (Thermo Scientific), 100 mg·L^−1^ l‐proline (Sigma), 1 μg·mL^−1^ hydrocortisone (Sigma), 0.6 μg·mL^−1^ insulin (Thermo Fisher Scientific), and 10 μm 1‐thioglycerol (Wako). After treatment with FTD alone, panitumumab alone, FTD/panitumumab combination, or DMSO for 24 h, the cells were lysed in ice‐cold lysis buffer [20 mm Tris/HCl [pH 7.4], 0.1% SDS, 1% NP‐40, 1 mm ethylenediaminetetraacetic acid, protease inhibitor cocktail (Sigma), and phosphatase inhibitors cocktail (Sigma)]. Equal amounts of protein from light and heavy samples were mixed, and the proteins were precipitated with five volumes of acetone. The precipitates were dissolved in 8 M urea, 100 mm triethylammonium bicarbonate (TEAB; Wako), and 5 mm tris(2‐carboxyethyl)phosphine (Thermo Scientific). The samples were digested with Lys‐C protease (Wako) at a ratio of 1:200 for 4 h, after which 50 mm iodoacetamide (Wako) was added for alkylation. The samples were diluted with 20 mm TEAB to 1 M urea concentration and then digested with sequencing‐grade modified trypsin (Promega) at a ratio of 1 : 100. The digested samples were acidified with 0.5% trifluoroacetic acid, and the supernatants were subsequently desalted on a C18 column (Shiseido C18MG, 4.6 × 250 mm, Tokyo, Japan). The desalted peptides were loaded onto TiO_2_ chips (GL Science, Tokyo, Japan) to enrich phosphopeptides in accordance with the instruction manual. The eluted phosphopeptides were desalted on a C18 column (Shiseido C18MG, 2.0 × 10 mm). The phosphopeptides were separated into 16 fractions on a polysulfoethyl A SCX column (PolyLC, 2.1 × 35 mm, 5 μm, 300 Å) using a gradient changing from buffer A [0.1% formic acid and 80% acetonitrile (MeCN)] to buffer B (350 mm ammonium formate, 30% MeCN, pH 3). The fractionated peptides were analyzed using fusion mass spectrometry (MS) (Thermo Scientific) coupled to a nano‐liquid chromatography (LC) system (EASY‐nLC 1000). The peptides were loaded onto a trap column (C18 Pepmap100, 3 μm, 0.075 × 20 mm) and separated on an analytical column (Reprosil‐Pur C18AQ, 3 μm, 0.075 × 150 mm; Nikkyo Technos, Tokyo, Japan) at a flow rate of 300 nL·min^−1^ for 90 min. LC/MS/MS measurements were performed by acquiring MS spectra at a resolution of 120 000 at 200 *m/z*, and data‐dependent higher‐energy collisional dissociation MS/MS at 30% normalized collision energy of the 30 most abundant ions in the ion trap. The dynamic exclusion time was 12 s. All MS raw files were processed to identify and quantify peptides with Proteome Discoverer 1.4 (Thermo Scientific) using mascot (v. 2.5, Matrix Science, London, UK) against the UniProt human protein database. The mass tolerances of a precursor and fragment were set to 10 ppm and 0.3 Da, respectively. A false discovery rate of 0.01 was applied to peptide identification.

### 2.6 Subcutaneous tumor xenograft models

All *in vivo* procedures were conducted in compliance with the Guide for the Care and Use of Laboratory Animals (8th Edition), US National Research Council, and approved by the Institutional Animal Care and Use Committee of the Shonan Research Center (#00011823), Takeda Pharmaceutical Company, Ltd. Female BALB/cA Jcl‐nu/nu (nude) mice and C.B17/Icr‐scid/scid Jcl (SCID) mice (CLEA, Tokyo, Japan) were maintained under specific pathogen‐free conditions. LIM1215 cells (5 × 10^6^) mixed with Matrigel were inoculated subcutaneously into the right flank of six‐ to seven‐week‐old SCID mice. Once established, the tumors were surgically excised, and smaller tumor fragments (about 2 mm in diameter) were subcutaneously implanted in the right flank of SCID mice. To establish the patient‐derived colon tumor xenograft (PDX) model, COL‐01‐JCK PDX line was obtained from the Central Institute for Experimental Animals (Kawasaki, Japan), and tumor fragments were implanted into the right flank of female nude mice. The mice were randomized when the mean tumor volume reached approximately 50–200 mm^3^. The mice were then treated with the vehicle (0.5% hydroxypropyl methylcellulose solution or saline), panitumumab (intraperitoneally), TAS‐102 [a mixture of FTD and TPI at a molar ratio of 1:0.5 (orally)], or panitumumab/TAS‐102 combination for 2 weeks. The tumor volumes were measured twice weekly with Vernier calipers and calculated as the length × width^2^ × 0.5. The treated/control ratio (T/C, %) was calculated by dividing the change in tumor volume in the drug‐treated mice by that in the vehicle‐treated control mice. The percentage of tumor regression was calculated as follows: tumor regression (%) at day *X* = [1−(tumor volume at day *X*/tumor volume at day 0)] × 100. Statistical comparisons of tumor volumes and body weights were made using Dunnett's multiple comparison tests; *P *<* *0.05 was considered statistically significant.

## 3 Results

### 3.1 The combination of panitumumab and FTD has significant antiproliferative effects in colon cancer cells

**Figure 1:**
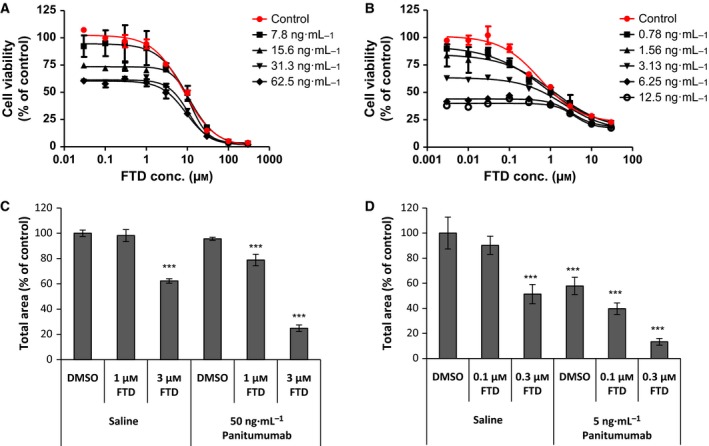
Panitumumab interacts with FTD to inhibit the growth of colon cancer cells. (A, B) Viability of SW48 (A) and LIM1215 (B) cells after cotreatment with panitumumab and FTD for 72 h. (C, D) Clonogenic assay was performed in triplicate for SW48 (C) and LIM1215 (D) cells. Total colony areas were individually determined using image analysis. Values represent the mean colony area (% of control). Error bars, standard deviation. Statistical comparisons of colony area were made using Dunnett's multiple comparison tests. Asterisks denote *P *<* *0.001 (***) versus the value for control colony area treated with DMSO and saline.

First, we evaluated the *in vitro* antiproliferative effects of panitumumab and FTD combination in SW48 and LIM1215 cells, which harbor the wild‐type *KRAS* and *BRAF* genes (Fig. 1A,B). Panitumumab blocked SW48 and LIM1215 cell proliferation in a dose‐dependent manner, although the maximum inhibition rates remained around 40 and 60%, respectively. FTD significantly inhibited proliferation of SW48 and LIM1215 cells with IC_50_ values of 8.1 and 0.57 μm, respectively. Cotreatment with FTD and panitumumab produced synergistic combination effects in SW48 cells (nonlinear blending score >20) and additive combination effects in LIM1215 cells (0.7 < CI < 1.3). Combination effects were also seen between FTD and cetuximab, another anti‐EGFR antibody, in LIM1215 cells but not in WiDr cells harboring the *BRAF* V600E mutation (Fig. S1). In clonogenic assays, cotreatment with panitumumab/FTD significantly suppressed colony formation and growth of SW48 and LIM1215 cells (Figs 1C,D and S2). Quantification analysis revealed that SW48 cell colony areas decreased by 75% when treated with 3 μm FTD and 50 ng·mL^−1^ panitumumab, while those of LIM1215 decreased by 87% at even lower concentrations, that is, 0.3 μm FTD and 5 ng·mL^−1^ panitumumab. FTD showed a broad spectrum of anticancer activities against various colon cancer cell lines, irrespective of *KRAS* or *BRAF* mutation status, with IC_50_ values ranging from single to low double‐digit micromolar concentrations (Fig. S3). LIM1215 cells were highly sensitive to FTD compared to other colon cancer cells (Fig. 1B). Therefore, LIM1215 cells were used to investigate the interaction between panitumumab and FTD further.

### 3.2 The combination of panitumumab and FTD leads to tumor regression in subcutaneous colon cancer xenograft models

**Figure 2:**
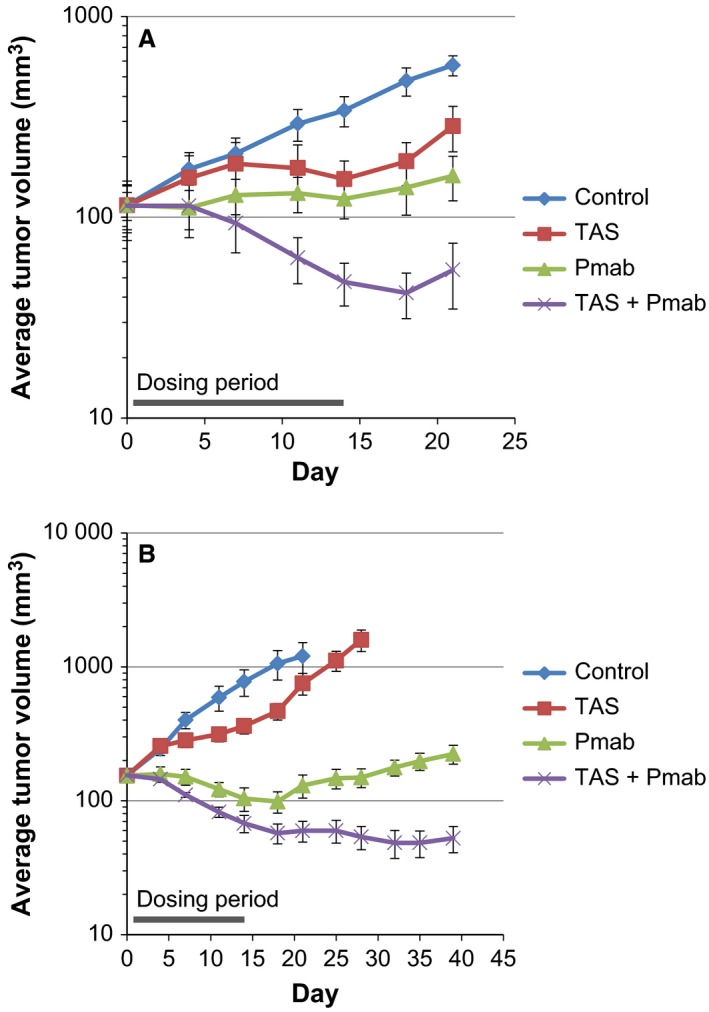
Coadministration of panitumumab (Pmab) and TAS‐102 induces tumor regression *in vivo*. Antitumor effects of Pmab, TAS‐102, and Pmab/TAS‐102 combination on the growth of subcutaneous LIM1215 (A) and COL‐01‐JCK (B) tumor xenografts. The mice were orally administered Pmab (3 mg·kg^−1^, twice weekly; intraperitoneally), TAS‐102 (75 mg·kg^−1^, twice daily on a 5‐days‐on/2‐days‐off schedule; orally), or a combination of both agents for 2 weeks (from days 1 to 14). The data represent the mean tumor volume ± standard error of the mean (*n *=* *5).

Colon cancer xenograft mouse models were used to evaluate the combination effects of panitumumab/TAS‐102 *in vivo*. SCID mice were inoculated subcutaneously with LIM1215 cells. After the tumors reached appropriate volumes, the mice received panitumumab (3 mg·kg^−1^, twice weekly; intraperitoneally), TAS‐102 (75 mg·kg^−1^, twice daily on a 5‐days‐on/2‐days‐off schedule; orally), their combination, or the vehicle for 2 weeks. As shown in Fig. 2A, treatment with panitumumab alone and TAS‐102 alone resulted in statistically significant tumor growth suppression with T/C values of 3.8 and 17.9%, respectively, on day 14 (*P *<* *0.001). The combination treatment had more profound antitumor effects, leading to substantial tumor regression, with a maximum regression rate of 63.2% on day 18. In this model, the mean body weight of the vehicle‐treated mice decreased gradually over the experimental period (Fig. S4A). However, two‐week treatments with panitumumab, TAS‐102, or the combination were tolerated and caused less body weight loss than vehicle treatment on day 21. To extend these findings, we conducted a similar efficacy study using a COL‐01‐JCK PDX model. COL‐01‐JCK is a colon PDX line without *KRAS* and *BRAF* mutations. TAS‐102 moderately inhibited tumor growth in this model, with the lowest T/C value of 33.4% on day 14 (*P *<* *0.01; Fig. 2B). In contrast, panitumumab treatment led to significant regression of tumor xenografts during the treatment period (by 36.1% on day 18 *vs*. day 0). Combination of panitumumab and TAS‐102 resulted in greater tumor regression than panitumumab alone, and the regression continued for more than 3 weeks after drug withdrawal (by 68.7% on day 35 *vs*. day 0). Although body weight loss was observed in the vehicle‐treated mice in this PDX model as well, all drug treatments were generally tolerated (Fig. S4B).

In addition, the effect of panitumumab on FTD incorporation into DNA was examined with an anti‐5‐bromo‐2‐deoxyuridine (BrdU) antibody because FTD incorporated into DNA can be recognized by BrdU antibodies (Kitao *et al*., 2016). Immunohistochemical staining experiments showed that there was no statistically significant difference in the percentage of FTD‐positive nuclei in tumor xenografts between the mice treated with FTD alone and those treated with FTD/panitumumab combination (Fig. S5).

### 3.3 Panitumumab blocks FTD‐induced ERK and AKT activation as well as EGFR gel mobility shift

**Figure 3:**
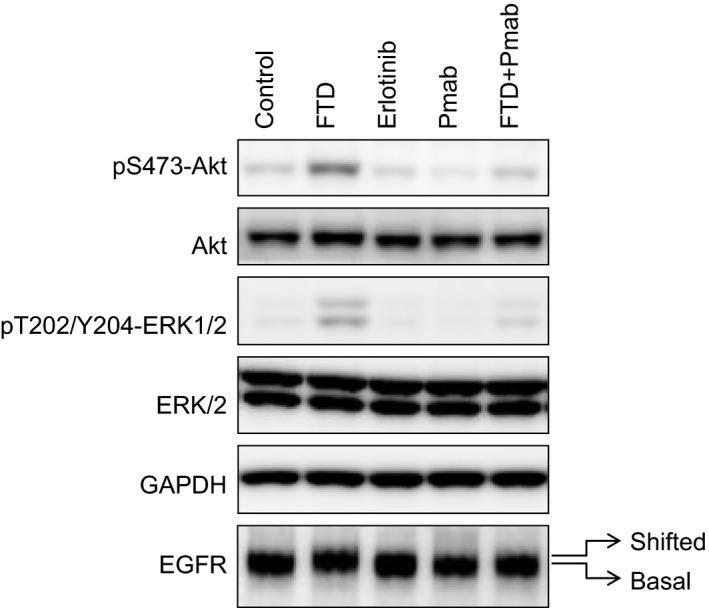
Panitumumab reverses FTD‐induced ERK1/2 and AKT phosphorylation and EGFR gel mobility shift in LIM1215 cells. LIM1215 cells were seeded onto a six‐well plate and cultured for 24 h. The cells were subsequently treated with FTD (3 μm), panitumumab (100 ng·mL ^−1^), erlotinib (10 μm), or a combination of FTD and panitumumab for 24 h, followed by western blotting analyses. FTD‐induced band mobility shift of EGFR is indicated as ‘shifted’ versus ‘basal’. GAPDH was used as a loading control. The blots are representative of two independent experiments.

To determine the potential interaction between panitumumab and FTD in EGFR signaling, we analyzed the phosphorylation status of signaling mediators ERK and AKT in FTD‐treated colon cancer cells using western blotting. Consistent with the results of a previous study (Bijnsdorp *et al*., 2010), ERK1/2, AKT, and STAT3 phosphorylation was induced in SW48 and LIM1215 cells after exposure to 3 μm FTD for 16 h or longer (Fig. S6). We tested whether panitumumab affected FTD‐induced phosphorylation of ERK1/2 and AKT and observed that cotreatment of panitumumab with FTD for 24 h suppressed FTD‐induced AKT and ERK phosphorylation (Fig. 3). Notably, FTD treatment also led to a slight EGFR gel mobility shift, suggesting that it modified EGFR to a certain extent. However, this EGFR mobility shift was inhibited by cotreatment with panitumumab.

### 3.4 FTD induces serine/threonine but not tyrosine phosphorylation of EGFR

We performed SILAC‐based phosphoproteomics analysis to investigate FTD‐induced EGFR modification and cellular signaling activation further. A complete list of phosphopeptides is provided in Table S3. The results of pathway analyses based on proteomic data confirmed the pharmacodynamic effects of FTD and panitumumab in LIM1215 cells. FTD treatment led to the significant enrichment of several Kyoto Encyclopedia of Genes and Genomes pathways associated with DNA damage, such as the Fanconi anemia pathway, cell cycle, homologous recombination, and p53 signaling (Table S1). Panitumumab treatment decreased the phosphopeptides derived from MAPK1 (ERK2), MAPK3 (ERK1), and ribosomal protein S6 kinases A1 and A3, suggestive of EGFR signaling inhibition by panitumumab (Tables 1 and S2).

**Table 1:** Effects of FTD on EGFR‐, ERK1/2‐, and p38 MAPK‐derived phosphopeptides. Phosphopeptides derived from EGFR, ERK1/2, and p38 MAPK were identified by phosphoproteomics analysis in LIM1215 cells treated with DMSO (ctr), FTD, panitumumab (Pmab), or a combination of FTD and panitumumab (FTD + Pmab). Amino acid numbers correspond to those in the UniProt protein database, except those for EGFR, which is a mature form, with the first 24 amino acids of the signal peptide cleaved off. Log_2_FC, log_2_ fold change

Name	Gene Symbol	Phosphosite	Log_2_FC FTD/ctr	Log_2_FC Pmab/ctr	Log_2_FC (FTD + Pmab)/ctr	Sequence	Start	End
EGFR	*EGFR*	pT669	1.34	−1.02	−0.35	ELVEPL[pT]PSGEAPNQALLR	663	681
		pS967	1.07	−0.86	0.32	MHLP[pS]PTDSNFYR	963	975
		pS1002	0.02	−0.31	0.20	ALMDEEDMDDVVDADEYLIPQQGFFS[pS]PSTSR	976	1007
		pS1004	0.05	−0.29	−0.08	ALMDEEDMDDVVDADEYLIPQQGFFSSP[pS]TSR	976	1007
		pS1015	1.06	0.07	1.15	TPLLSSL[pS]ATSNNSTVACIDR	1008	1028
		pS1018	1.07	−0.05	0.74	TPLLSSLSAT[pS]NNSTVACIDR	1008	1028
		pS1039 pS1021	0.78	0.17	1.14	TPLLSSLSAT[pS]NN[pS]TVACIDR	1008	1028
		pT1017 pS1018	1.46	0.21	1.40	TPLLSSLSA[pT][pS]NNSTVACIDR	1008	1028
		pS1040	−0.70	−0.74	−0.95	NGLQSCPIKED[pS]FLQR	1029	1044
		pS1047 pT1050	2.18	0.19	1.01	YS[pS]DP[pT]GALTEDSIDDTFLPVPEYINQSVPK	1045	1075
		pS1057	0.77	−0.58	0.01	YSSDPTGALTED[pS]IDDTFLPVPEYINQSVPK	1045	1075
		pY1068	−0.15	0.16	−0.13	YSSDPTGALTEDSIDDTFLPVPE[pY]INQSVPK	1045	1075
		pS1142	0.53	−0.78	−0.45	GSHQI[pS]LDNPDYQQDFFPK	1137	1155
		pY1148	−0.09	0.09	−0.28	GSHQISLDNPD[pY]QQDFFPK	1137	1155
		pY1173	−0.08	0.01	−0.30	GSTAENAE[pY]LR	1165	1175
ERK1	*MAPK3*	pT202 pY204	0.77	−1.74	−1.91	IADPEHDHTGFL[pT]E[pY]VATR	190	208
		pY204	0.59	−0.78	−0.89	IADPEHDHTGFLTE[pY]VATR	190	208
ERK2	*MAPK1*	pT185 pY187	0.75	−2.08	−1.96	VADPDHDHTGFL[pT]E[pY]VATR	173	191
		pY187	0.55	−0.83	−1.07	VADPDHDHTGFLTE[pY]VATR	173	191
p38 alpha	*MAPK14*	pT180	0.69	0.41	1.19	HTDDEM[pT]GYVATR	174	186
		pT180 pY182	0.56	0.75	1.51	HTDDEM[pT]G[pY]VATR	174	186
		pY182	0.82	0.35	0.70	HTDDEMTG[pY]VATR	174	186
p38 delta	*MAPK13*	pT180	1.04		0.76	HADAEM[pT]GYVVTR	174	186
		pY182	0.86	0.40	1.04	HADAEMTG[pY]VVTR	174	186

**Figure 4:**
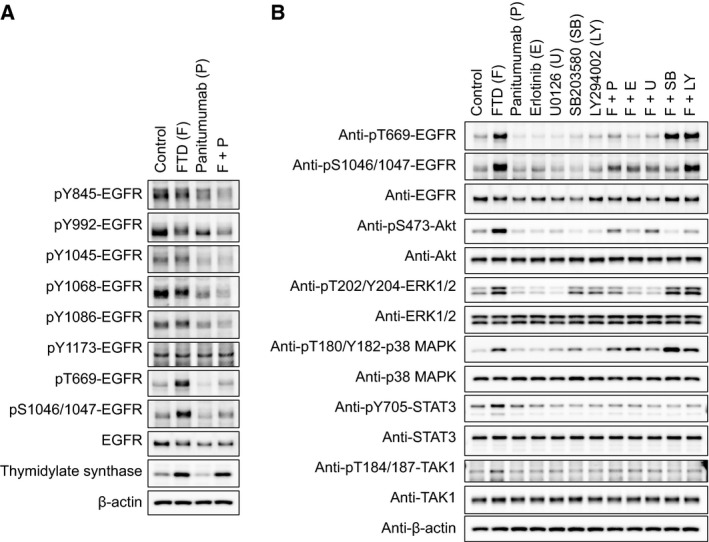
The effects of panitumumab and other kinase inhibitors on FTD‐induced phosphorylation of EGFR and intracellular kinases in LIM1215 cells. LIM1215 cells were seeded onto six‐well plates and cultured for 24 h. (A) EGFR phosphorylation at specific residues was probed with each phospho‐specific EGFR antibody following a 24‐h treatment with FTD (3 μm), panitumumab (100 ng·mL ^−1^), or their combination. Induction of thymidylate synthase expression was used as a marker of pharmacodynamic response to FTD. β‐Actin was used as a loading control. (B) LIM1215 cells were treated for 24 h with FTD (3 μm), panitumumab (100 ng·mL ^−1^), the EGFR tyrosine kinase inhibitor erlotinib (1 μm), MEK inhibitor U0126 (10 μm), p38 MAPK inhibitor SB203580 (10 μm), PI3K inhibitor LY294002 (10 μm), or their combinations as indicated.

As FTD‐induced gel mobility shift of EGFR occurred in parallel with AKT and ERK phosphorylation and was reversed by cotreatment with panitumumab, we speculated that FTD might have induced EGFR tyrosine phosphorylation. However, SILAC‐based analyses revealed that FTD‐induced EGFR phosphorylation occurred at serine/threonine residues rather than at tyrosine residues (Table 1). Consistently, western blotting confirmed that FTD stimulated EGFR phosphorylation at threonine (T) 669 and serine (S) 1046/1047, but not at known tyrosine phosphorylation sites (Fig. 4A). The time dependence and concentration dependence of FTD‐induced EGFR serine/threonine phosphorylation were confirmed in additional experiments (Figs S7 and S8). Panitumumab alone reduced the basal levels of EGFR tyrosine phosphorylation at almost all sites tested (Fig. 4A). Moreover, cotreatment with panitumumab suppressed FTD‐induced serine/threonine phosphorylation of EGFR. Increase in TS protein level, a pharmacodynamic marker of inhibition of TS activity by fluoropyrimidine derivatives (Chu *et al*., 1991), was observed with a similar extent in cells treated with both FTD alone and FTD/panitumumab combination. These results suggest that cotreatment with panitumumab has little effects of FTD on TS inhibition, although TS inhibition is not considered to be the main mechanism underlying FTD cytotoxicity.

### 3.5 Serine/threonine phosphorylation of EGFR is dependent on the activation of MEK/ERK signaling pathway

To investigate the mechanism underlying FTD‐induced serine/threonine phosphorylation of EGFR, LIM1215 cells were cotreated with FTD and several kinase inhibitors (Fig. 4B). FTD‐induced EGFR serine/threonine phosphorylation was suppressed by erlotinib and panitumumab. It was also blocked by the MEK inhibitor U0126. The PI3K inhibitor LY294002 inhibited FTD‐induced AKT/STAT3 phosphorylation, but not FTD‐induced ERK1/2 and EGFR serine/threonine phosphorylation. In addition, the time dependence and concentration dependence between FTD‐induced ERK1/2 phosphorylation and EGFR serine/threonine phosphorylation (Figs S7 and S8) were similar. Using the SILAC‐based phosphoproteomic data, we determined the responsible kinases and their substrates that were affected by FTD treatment and created a kinase–substrate connected network (Fig. S9). A subnetwork of EGFR and the first neighbors showed significant contributions of ERK1/2 (MAPK3 and MAPK1, respectively) to FTD‐induced serine/threonine phosphorylation of EGFR. These results suggest that EGFR serine/threonine phosphorylation occurs downstream of MEK/ERK signaling pathway.

Trifluridine also induced p38 MAPK phosphorylation (Fig. 4B, Table 1), which was not affected by either panitumumab or erlotinib, suggesting that upstream signaling through p38 MAPK phosphorylation and ERK/AKT/STAT3 phosphorylation was differentially induced by FTD. However, FTD‐induced AKT/STAT3 and EGFR S1046/1047 phosphorylation was inhibited by the p38 MAPK inhibitor SB203580, suggestive of signaling crosstalk.

### 3.6 Cotreatment with a MEK inhibitor and FTD shows additive antiproliferative effects

**Figure 5:**
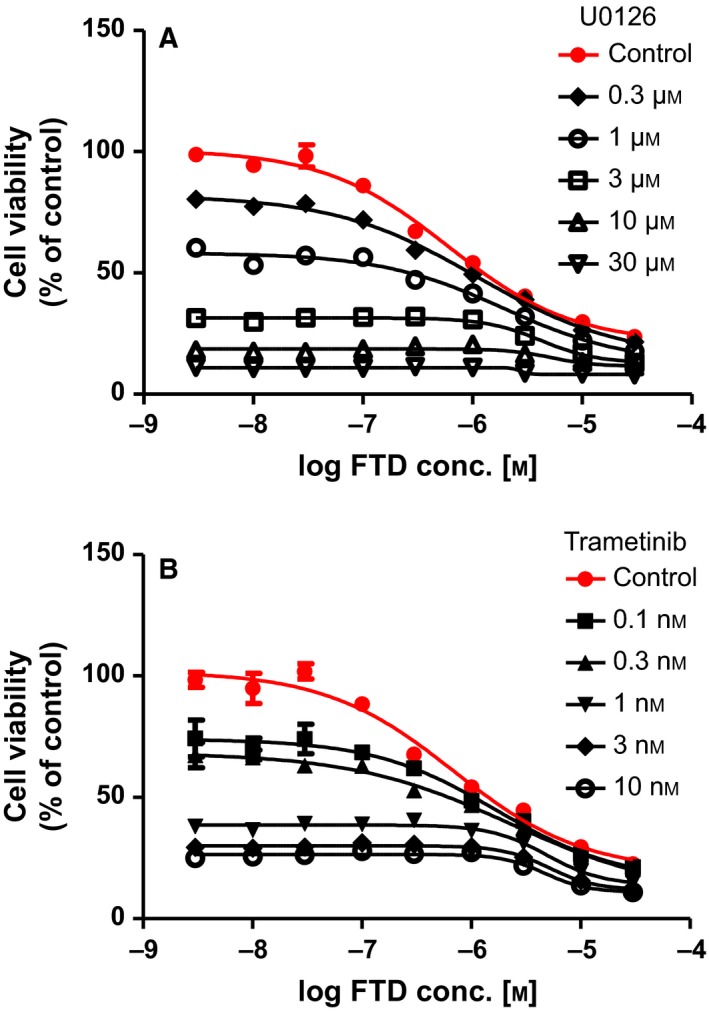
Combination treatment of FTD with MEK inhibitors shows additive antiproliferative effects in LIM1215 cells. The viability of LIM1215 cells was determined after cotreatment with FTD and U0126 (A) or FTD and trametinib (B) for 72 h.

As the MEK inhibitor U0126 blocked FTD‐induced AKT/ERK/STAT3, EGFR T669, and S1046/1047 phosphorylation to a similar extent as panitumumab, the effect of cotreatment with FTD and the MEK inhibitors U0126 or trametinib on cell proliferation was evaluated. Cotreatment with either FTD/U0126 or FTD/trametinib yielded additive antiproliferative effects in LIM1215 cells (0.7 < CI< 1.3; Fig. 5).

## 4 Discussion

In this study, we demonstrated that the combination treatment of TAS‐102 and panitumumab exerted significant anticancer activity compared to that achieved by single‐agent treatment in *in vitro* and *in vivo* wild‐type *KRAS* colon cancer models. Previous studies suggested that TAS‐102 may potentially enhance the effects of combination treatment of chemotherapeutics irinotecan and oxaliplatin (Nukatsuka *et al*., 2015; Temmink *et al*., 2007b) or targeted therapeutics, such as bevacizumab and anti‐EGFR agents (Tsukihara *et al*., 2015; Bijnsdorp *et al*., 2010). Our results are consistent with that of Tsukihara *et al*. (2015), wherein TAS‐102/panitumumab combination suppresses tumor growth in an SW48 tumor xenograft model. However, the *in vivo* combination efficacy was prominent in LIM1215 and COL‐01‐JCK models used in this study when compared with that in SW48 model, as reflected by the profound and sustained tumor regression achieved with a similar dosing regimen. The difference in responses among these wild‐type *KRAS* colon cancer models may provide an intriguing tool for exploring determinants or predictive markers of the response. In our two models, the vehicle‐treated mice experienced gradual body weight loss as the tumors grew, which was probably due to cancer‐related cachexia. However, the combination regimen was tolerated and had no confounding effects on body weight loss caused by TAS‐102, suggesting that TAS‐102 and panitumumab had few overlapping toxicities. Indeed, the most frequently observed adverse events associated with TAS‐102 in a phase III study were neutropenia and leukopenia (Mayer *et al*., 2015), while those associated with panitumumab were skin toxicities, hypomagnesemia, and diarrhea (Van Cutsem *et al*., 2007). However, panitumumab has no cross‐reactivity with mouse EGFR, which makes it difficult to assess the therapeutic window in tumor xenograft models. Thus, careful evaluation of safety is needed in clinical settings.

We also assessed the molecular mechanism underlying the interaction between FTD and panitumumab, and found that FTD treatment induced ERK1/2, AKT, and STAT3 phosphorylation in SW48 and LIM1215 cells. Several other chemotherapeutics induced similar ERK/AKT/STAT3 activation, which is considered to mediate prosurvival signaling and be implicated in resistance to these genotoxic agents (McCubrey *et al*., 2011; Liu *et al*., 2014; Winograd‐Katz and Levitzki, 2006; Mabuchi *et al*., 2002; Taylor *et al*., 2011; Poli and Camporeale, 2015). Thus, we believe that FTD‐induced activation of ERK/AKT/STAT3 plays a similar role in the adaptive response of colon cancer cells to genotoxic stress caused by FTD. In particular, the MEK inhibitors U0126 and trametinib when combined with FTD caused additive effects on the proliferation of LIM1215 cells. Therefore, we believe that MEK/ERK signaling may, at least partly, mediate prosurvival signaling in response to FTD.

We further observed that FTD‐induced ERK/AKT/STAT3 phosphorylation was suppressed by panitumumab and erlotinib. Initially, these results led us to speculate that FTD could induce EGFR tyrosine kinase activation and subsequent phosphorylation of its downstream molecules. However, SILAC‐based proteomics and western blotting revealed that FTD had no effects on EGFR tyrosine phosphorylation status. Instead, FTD induced EGFR serine/threonine phosphorylation, which was reversed by combination treatment with panitumumab, erlotinib, or the MEK inhibitor U0126. Therefore, we proposed a model in which the basal activity of EGFR tyrosine kinase is required for FTD‐induced ERK/AKT/STAT3 phosphorylation, and in which EGFR serine/threonine phosphorylation is a downstream event of MEK/ERK signaling. This model is also supported by kinase–substrate connected network analysis based on phosphoproteomics data, which indicates an important contribution of ERK1/2 to FTD‐induced EGFR serine/threonine phosphorylation. Consistent with these data, prior studies have implicated threonine 669 of EGFR as an ERK phosphorylation site (Li *et al*., 2008; Takishima *et al*., 1991; Northwood *et al*., 1991).

The significance of FTD‐induced EGFR serine/threonine phosphorylation, however, remains to be elucidated. Nishimura *et al*. (Nishimura *et al*., 2009) showed that tumor necrosis factor alpha (TNF‐α)‐induced EGFR phosphorylation at T669 and S1046/1047 stimulated EGFR endocytosis, leading to the survival of cells exposed to TNF‐α receptor death signal. Further, Winograd‐Katz and Levitzki (Winograd‐Katz and Levitzki, 2006) proposed that cisplatin‐induced EGFR T669 phosphorylation similarly increased EGFR endocytosis, which might switch signaling pathways from proliferation to survival. Thus, it is of interest to further investigate whether FTD‐induced EGFR serine/threonine phosphorylation mediates similar prosurvival signaling through EGFR endocytosis in colon cancer cells. One possible approach is to introduce mutations that prevent phosphorylation by substitution of serine/threonine residues of EGFR and evaluate FTD sensitivity of the cells with these mutant EGFRs.

**Figure 6:**
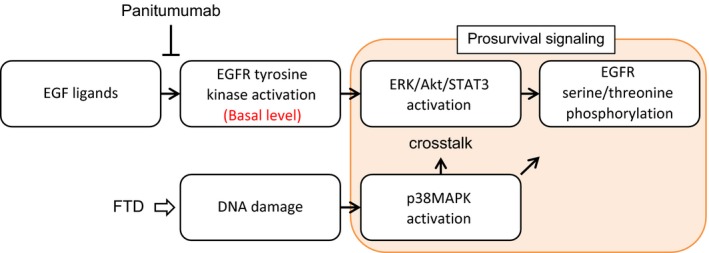
Schematic diagram of FTD‐induced prosurvival signals. FTD induces DNA damage, which in turn elicits p38 MAPK activation. p38 MAPK signaling interacts with the signaling mediated by the basal activity of the EGFR tyrosine kinase through ERK/AKT/STAT3, promoting prosurvival signaling. EGFR serine/threonine phosphorylation occurs downstream of ERK/AKT/STAT3. Panitumumab inhibits the basal activity of EGFR, subsequent ERK/AKT/STAT3 activation, and EGFR serine/threonine phosphorylation, thereby making cells more sensitive to FTD‐induced DNA damage.

We also observed that FTD treatment induced phosphorylation of p38 MAPK. p38 MAPK phosphorylation is induced by a diverse set of intra‐ and extracellular stimuli, including genotoxic stress caused by chemotherapeutics such as cisplatin mediating prosurvival signaling (Winograd‐Katz and Levitzki, 2006). Unlike ERK/AKT/STAT3 phosphorylation, FTD‐induced p38 MAPK phosphorylation was not significantly affected by panitumumab or erlotinib, suggesting that p38 MAPK phosphorylation was independent of EGFR tyrosine kinase activity. However, pharmacological inhibition of p38 MAPK decreased FTD‐induced AKT, STAT3, and EGFR S1046/1047 phosphorylation. These results suggest that there is a crosstalk between p38 MAPK and EGFR/AKT/STAT3 signaling. Accordingly, we proposed a model in which FTD‐induced p38 MAPK activation and EGFR‐dependent ERK/AKT/STAT3 activation cooperatively promote prosurvival signaling (Fig. 6).

In conclusion, we demonstrated that cotreatment with panitumumab and TAS‐102 had significant *in vitro* and *in vivo* anticancer effects in different colon cancer models. We also showed that panitumumab suppressed FTD‐induced ERK/AKT/STAT3 activation, which we believe is the mechanism underlying the combinatorial effects of panitumumab and FTD. These preclinical findings provide a compelling rationale for evaluating the efficacy of panitumumab in combination with TAS‐102 in a clinical setting. Currently, a phase I/II APOLLON study is under evaluation, which is designed to investigate the safety and efficacy of panitumumab in combination with TAS‐102 in patients with wild‐type *RAS* mCRC, who are refractory to standard chemotherapy (https://clinicaltrials.gov/ct2/show/NCT02613221).

## Author contributions

YB, TT, YS, KS, JS, and KN conceived and designed the project. YB, TT, YS, MG, HS, and SE acquired and analyzed the data. YB, KS, and KN wrote the manuscript. JS and KN reviewed, revised, and approved the final version of the manuscript. YB, TT, and YS contributed equally to the article. JS and KN share senior authorship of this article.

## Conflict of interest

All authors are current or former employees of Takeda Pharmaceutical Company Limited.

## Supporting information


**Fig. S1.** Cotreatment with cetuximab and FTD inhibits proliferation of LIM1215 cells but not WiDr cells.
**Fig. S2.** Panitumumab interacts with FTD to inhibit the clonogenic growth of colon cancer cells.
**Fig. S3.** FTD inhibits cell proliferation of various colon cancer cell lines, irrespective of the *KRAS* and *BRAF* mutation statuses.
**Fig. S4.** Body weight change in tumor‐bearing mice.
**Fig. S5.** Immunohistochemical staining for FTD incorporated into DNA in the LIM1215 tumor xenograft model.
**Fig. S6.** FTD‐induced phosphorylation of AKT, ERK1/2, and STAT3 in SW48 and LIM1215 cells.
**Fig. S7.** Time dependency of FTD‐induced AKT/ERK/STAT3 and EGFR serine/threonine phosphorylation.
**Fig. S8.** Concentration dependency of FTD‐induced AKT, ERK1/2, and EGFR serine/threonine phosphorylation.
**Fig. S9.** A highly connected subnetwork within EGFR and first neighbors.

**Appendix S1.** Supplementary materials and methods.

**Table S1.** List of genes from the KEGG pathways identified by phosphoproteomic analysis, showing the effects of FTD versus control in LIM1215 cells.
**Table S2.** List of genes from the KEGG pathways identified by phosphoproteomic analysis, showing the effects of panitumumab versus control in LIM1215 cells.

**Table S3.** A complete list of phosphopeptides in SILAC‐based phosphoproteomics analysis.
