# Supplementary material for: Panitumumab interaction with TAS‐102 leads to combinational anticancer effects via blocking of EGFR‐mediated tumor response to trifluridine
Source: Mol Oncol. 2017 May 30;11(8):1065–77. doi: 10.1002/1878-0261.12074 (PMC5537908; doi:10.1002/1878-0261.12074)

Supplementary Figure S1

A

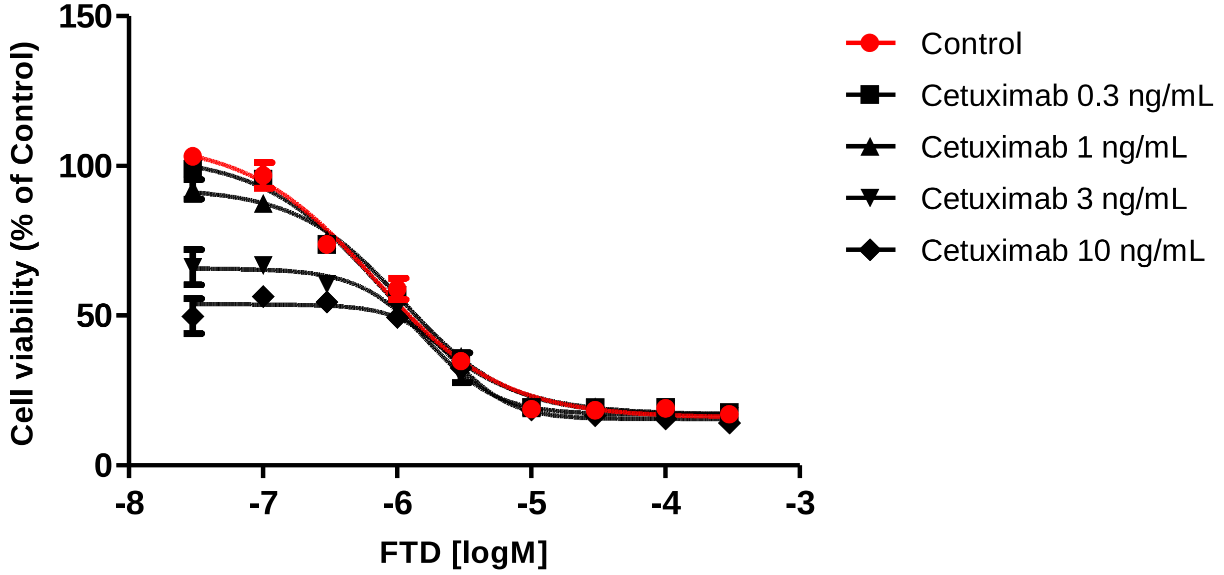

B

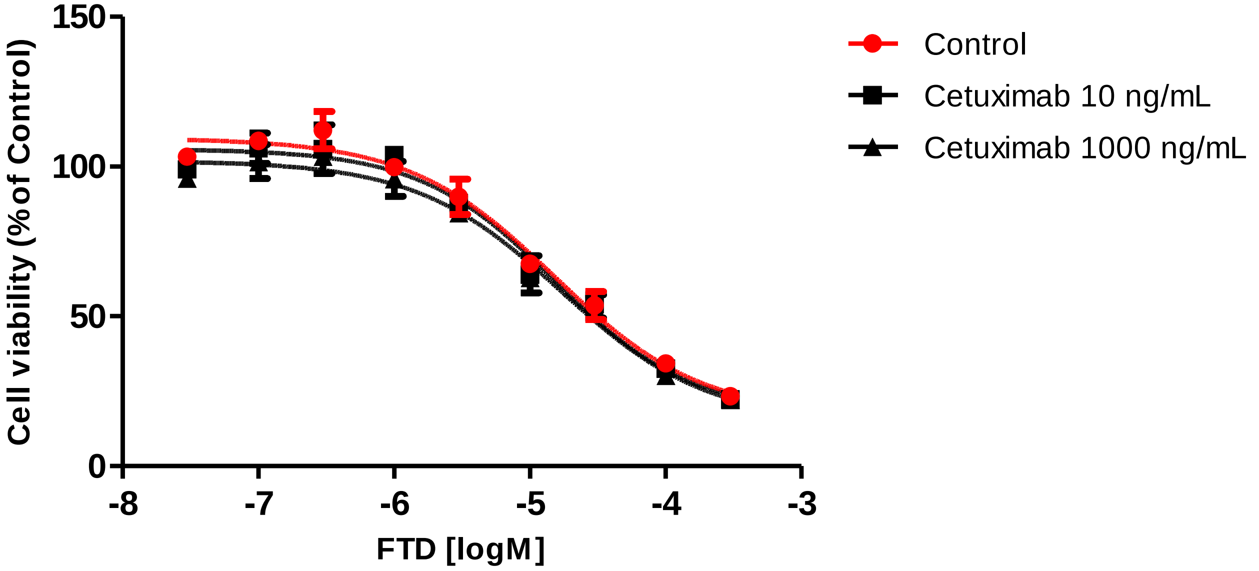

Supplementary Figure S2

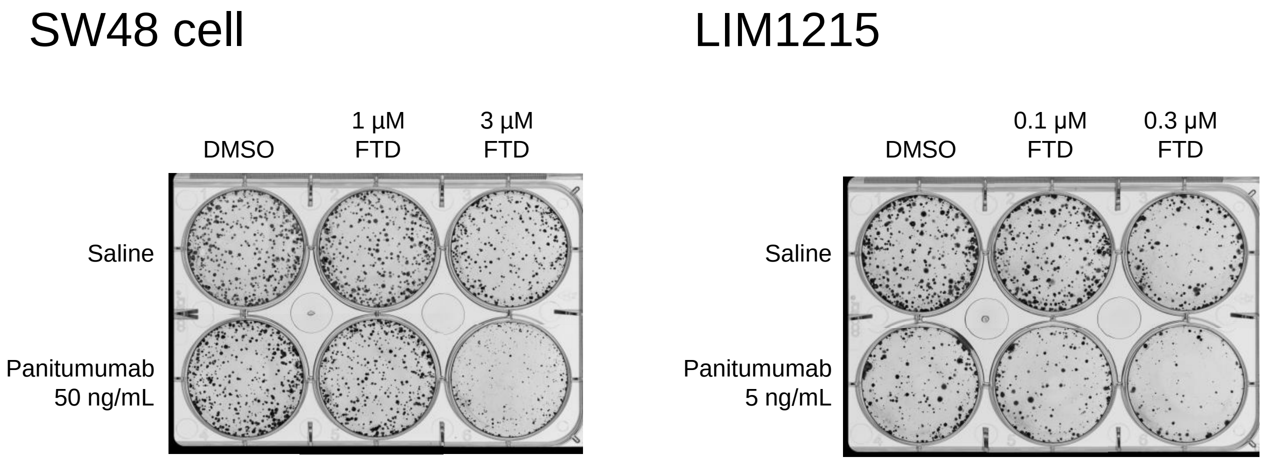

Supplementary Figure S3

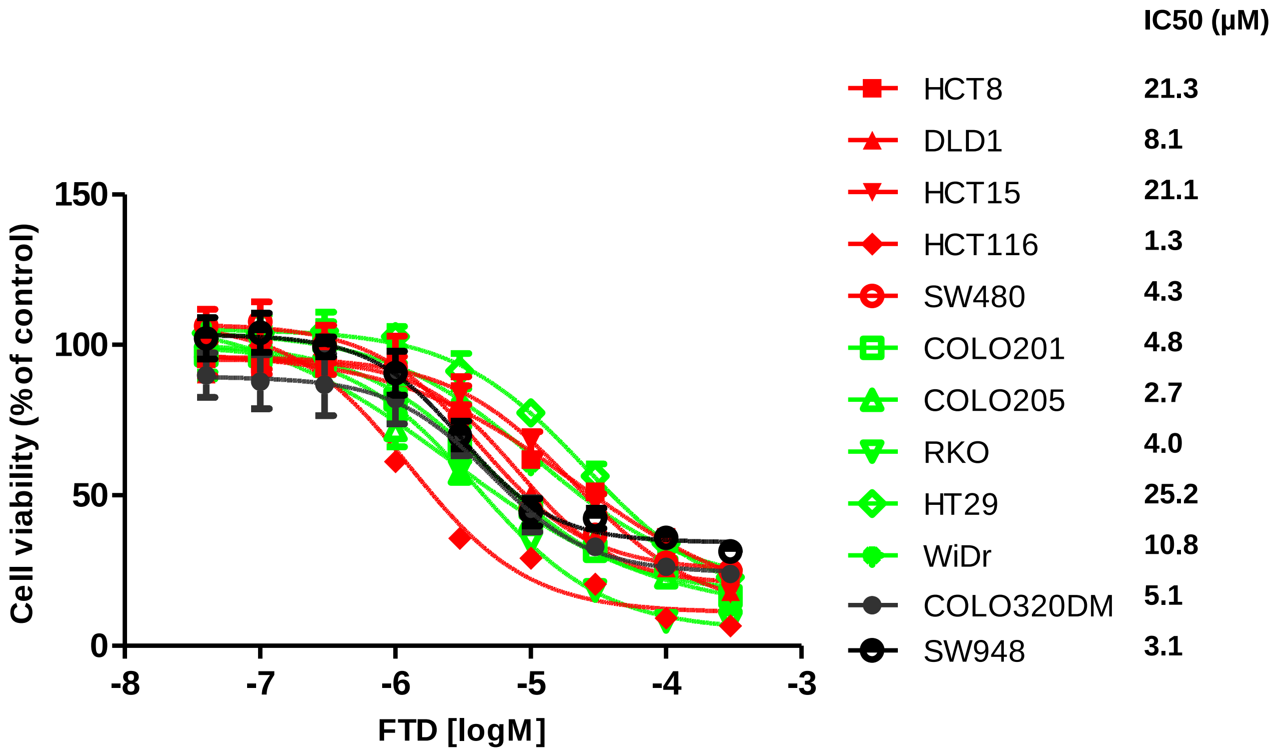

Supplementary Figure S4

A

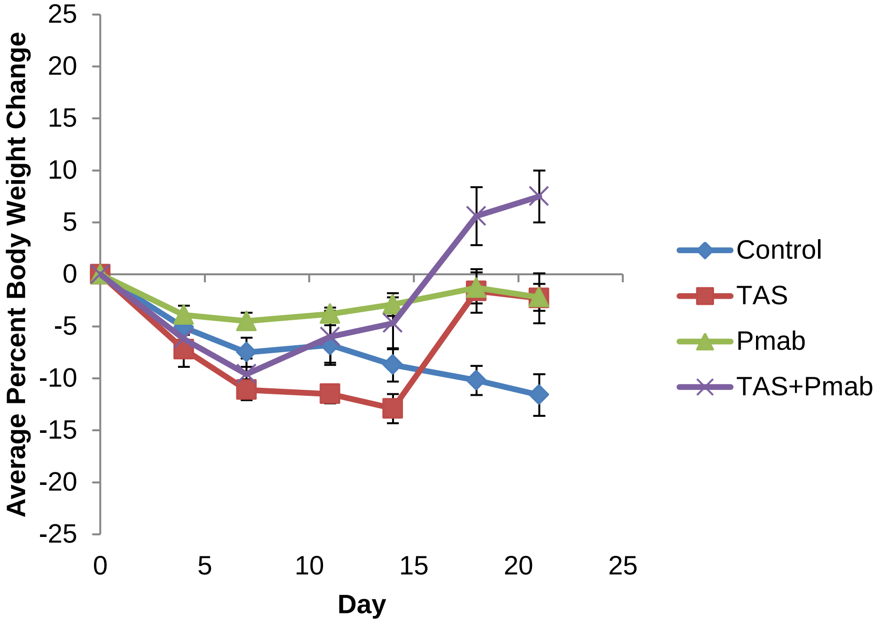

B

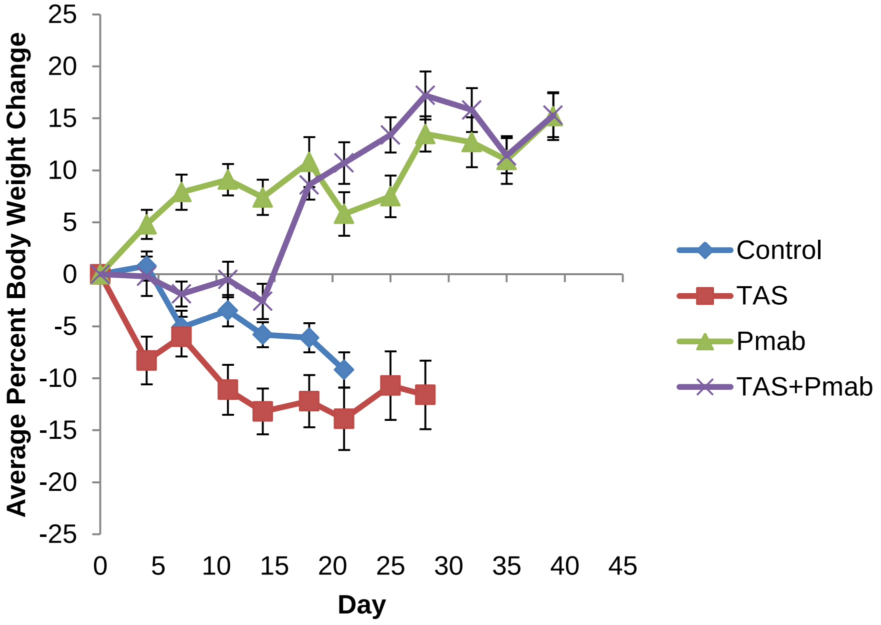

Supplementary Figure S5

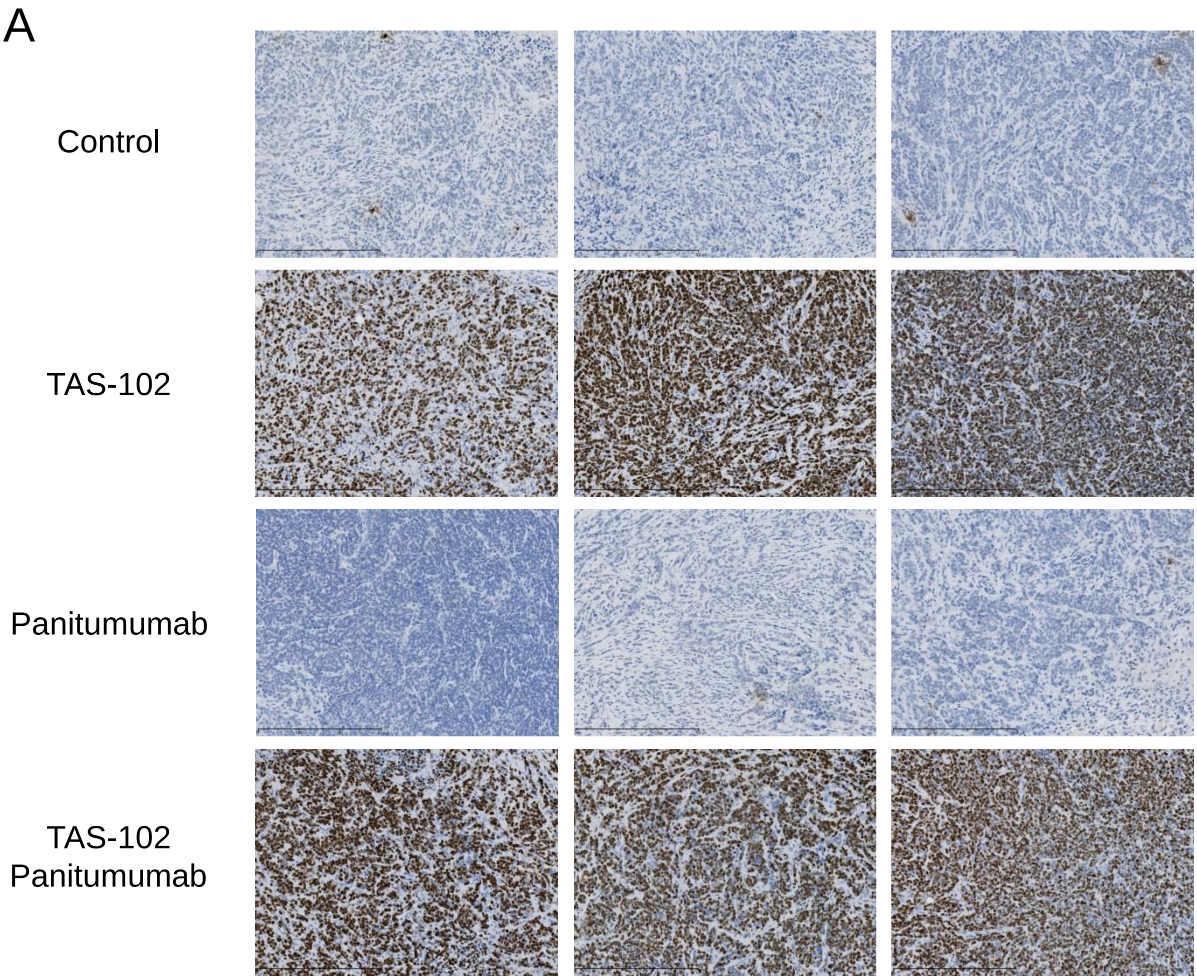

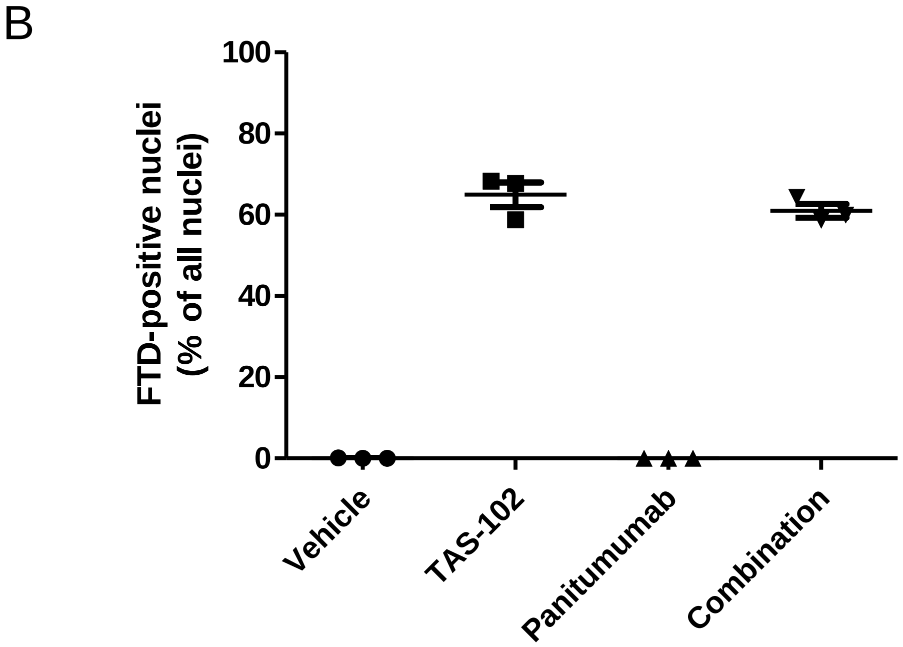

Supplementary Figure S6

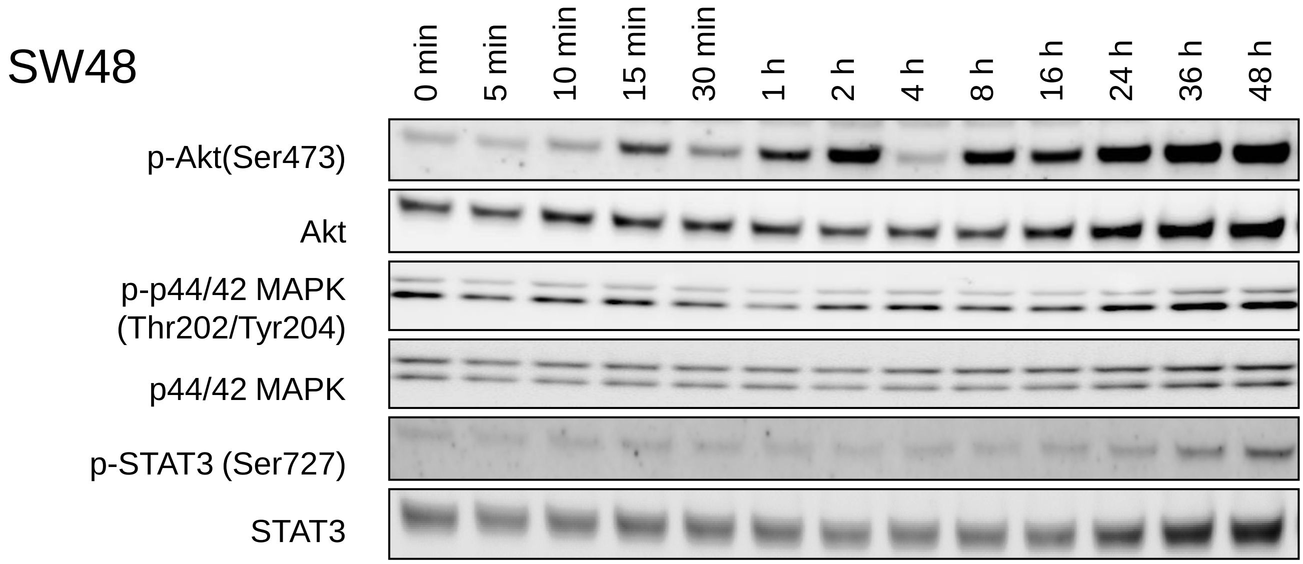

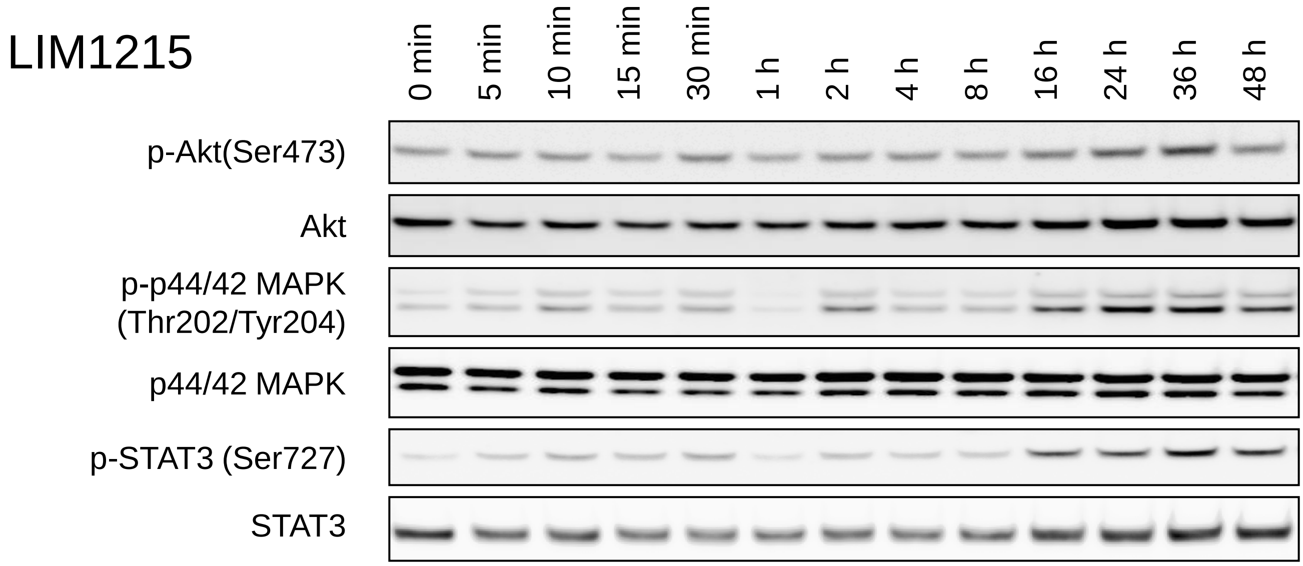

Supplementary Figure S7

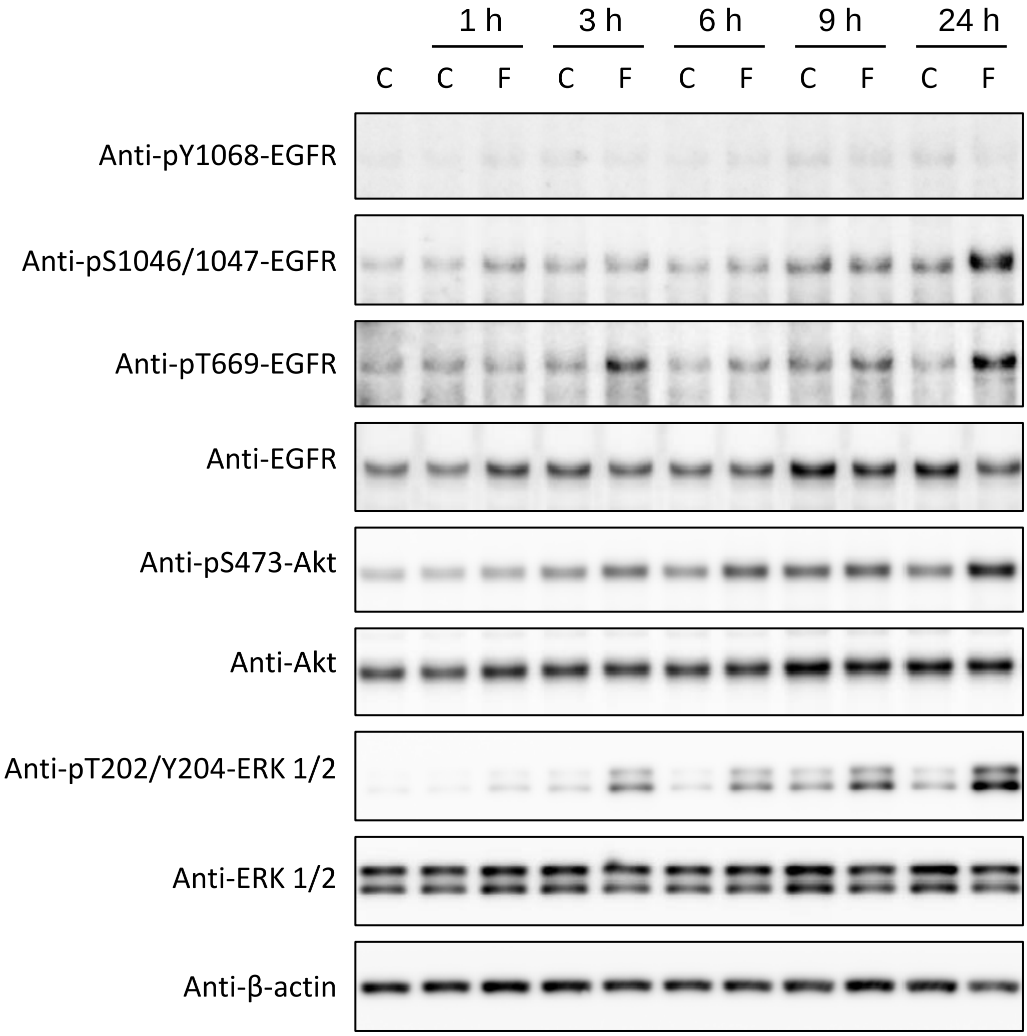

Supplementary Figure S8

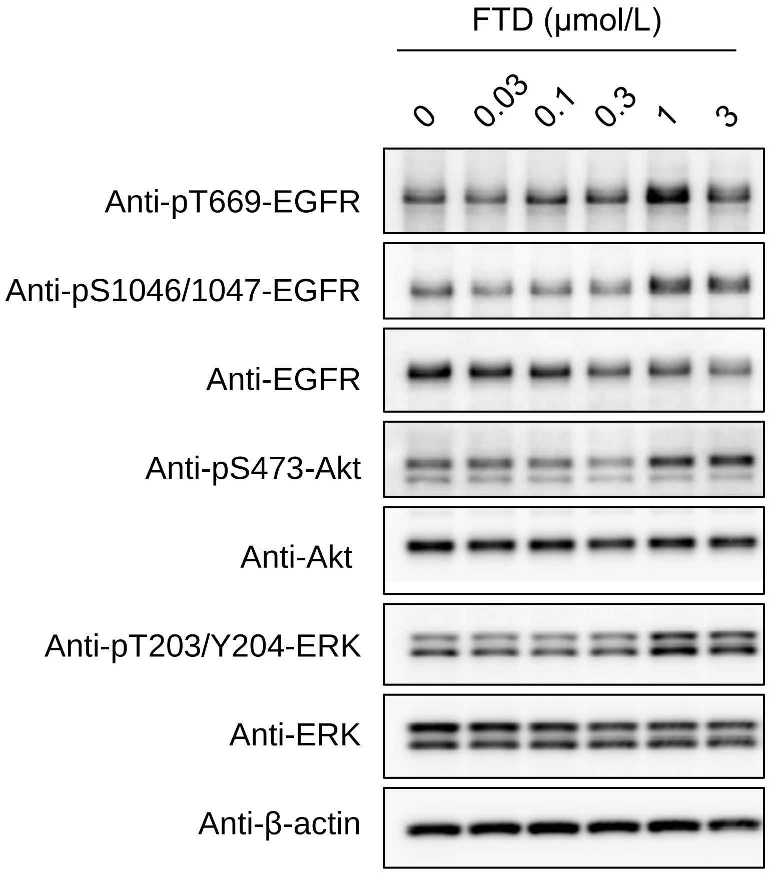

Supplementary Figure S9

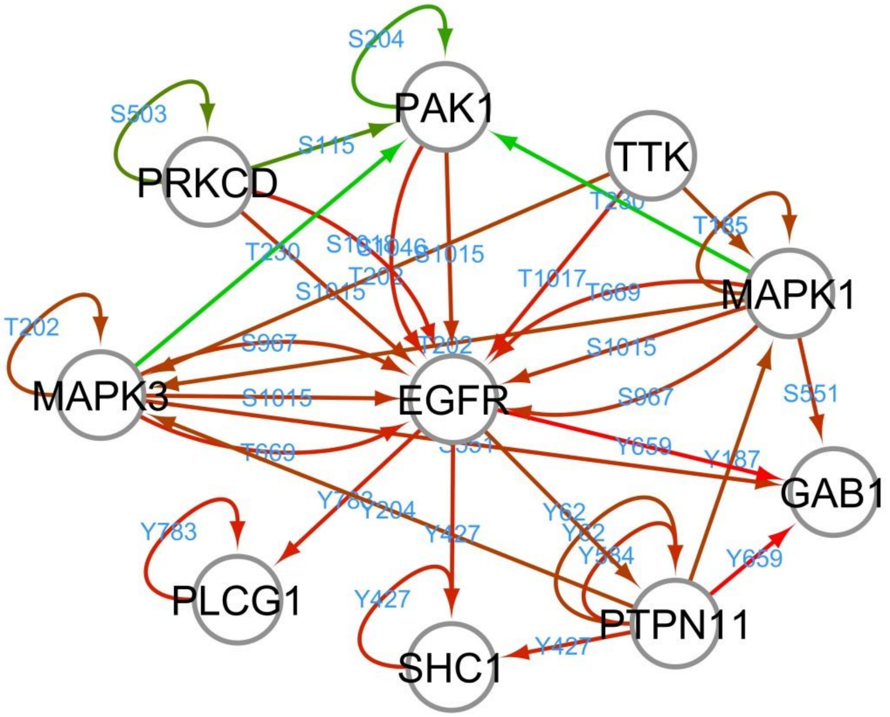

Supplement: Supplementary file 1 — Fig. S1. Cotreatment with cetuximab and FTD inhibits proliferation of LIM1215 cells but not WiDr cells. Fig. S2. Panitumumab interacts with FTD to inhibit the clonogenic growth of colon cancer cells. Fig. S3. FTD inhibits cell proliferation of various colon cancer cell lines, irrespective of the KRAS and BRAF mutation statuses. Fig. S4. Body weight change in tumor‐bearing mice. Fig. S5. Immunohistochemical staining for FTD incorporated into DNA in the LIM1215 tumor xenograft model. Fig. S6. FTD‐induced phosphorylation of AKT, ERK1/2, and STAT3 in SW48 and LIM1215 cells. Fig. S7. Time dependency of FTD‐induced AKT/ERK/STAT3 and EGFR serine/threonine phosphorylation. Fig. S8. Concentration dependency of FTD‐induced AKT, ERK1/2, and EGFR serine/threonine phosphorylation. Fig. S9. A highly connected subnetwork within EGFR and first neighbors. [file MOL2-11-1065-s001.pdf]
