# Supplementary material for: Panitumumab interaction with TAS‐102 leads to combinational anticancer effects via blocking of EGFR‐mediated tumor response to trifluridine
Source: Mol Oncol. 2017 May 30;11(8):1065–77. doi: 10.1002/1878-0261.12074 (PMC5537908; doi:10.1002/1878-0261.12074)
Supplement: Supplementary file 2 — Appendix S1. Supplementary materials and methods. [file MOL2-11-1065-s002.docx]

**Supplementary Information**

**Panitumumab interaction with TAS-102 leads to combinational anticancer effects via blocking of EGFR-mediated tumor response to trifluridine**

Yuji Baba, Toshiya Tamura, Yoshihiko Satoh, Masamitsu Gotou, Hiroshi Sawada, Shunsuke Ebara, Kazunori Shibuya, Jumpei Soeda, Kazuhide Nakamura

**Supplementary Materials and Methods**

**Cell culture and viability assay for colon cancer cell lines**

Human colon cancer cells, HCT-8, HCT-15, HCT-116, COLO-205 (ATCC), CW-2 (RIKEN, Saitama, Japan), and COLO-201 (JCRB, Osaka, Japan), were cultured in RPMI 1640 medium (Wako) with 10% fetal bovine serum (FBS; Thermo Scientific). WiDr and RKO cells (ATCC, Manassas, VA) were cultured in E-MEM (Wako) with 10% FBS, MEM non-essential amino acid solution (Wako), and 1 mM sodium pyruvate (Wako). HT-29 and DLD1 cells (Horizon Discovery) were cultured in McCoy's 5A medium (Wako) with 10% FBS. SW480, SW620, and SW948 cells (ATCC) were cultured in L15 medium (Thermo Scientific) with 10% FBS. COLO320DM cells (JCRB) were cultured in DMEM (Wako) with 10% FBS. For cell proliferation assays, colon cancer cells were plated in 96-well plates at appropriate densities. Twenty-four hours after cell plating, a serial dilution of FTD was added to culture media. Cells were then cultured for an additional 72 h, and cell viability was determined by the CellTiter-Glo assay (Promega).

**Immunohistochemical staining of paraffin-embedded samples**

Tumor-bearing mice, subcutaneously injected with LIM1215 cells, were administered the vehicle, TAS-102 (75 mg/mL, twice daily from day 1 to day 4), panitumumab (3 mg/mL, on days 1 and 4), or TAS-102/panitumumab. Tumor xenografts were excised on day 5, fixed with 10% neutral buffered formalin for 24 h, and embedded in paraffin. Sliced sections were deparaffinized with xylene, then rehydrated, and subjected to citrate buffer antigen retrieval (98 °C, 40 min). Immunohistochemical staining for FTD was performed as described previously (Kitao et al., 2016) using an anti-BrdU antibody (clone 3D4; BD Pharmingen, Franklin Lakes, NJ) and a peroxidase-conjugated anti-mouse antibody (Histofine Simplestain Max PO, Nichirei, Tokyo, Japan). Peroxidase activity was detected with diaminobenzidine. Sections were counterstained with hematoxylin, and stained images were acquired with a Nanozoomer digital slide scanner (Hamamatsu Photonics, Shizuoka, Japan). FTD-positive nuclei were automatically counted by setting a fixed threshold to remove the background, and the percentage of FTD-positive nuclei relative to all nuclei was determined with the image analysis software Tissue Studio (Definiens, Munich, Germany).

**Network analysis**

The NetworKIN (version 3.0) algorithm was used for the prediction of potential kinase–substrate relationships (Horn et al., 2014). The NetworKIN algorithm combines network-proximity scores for protein–protein interactions and NetPhorest probabilities on the basis of network distances and peptide sequences, respectively. Phosphopeptides with fold changes of at least 1.5 in duplicate samples after treatment with FTD alone were selected as altered phosphopeptides. For network analysis, the kinase–substrate relationships of the altered phosphopeptides were filtered to include only those with a NetworKIN score of >2, and kinases that altered phosphopeptides were selected. Cytoscape (version 3.4.0) was used for network data integration, analysis, and visualization for the selected kinases and substrates (Shannon et al., 2003).

**Pathway enrichment analysis**

Kyoto Encyclopedia of Genes and Genomes (KEGG) enriched pathways were analyzed using the Database for Annotation, Visualization, and Integrated Discovery (DAVID) Bioinformatics Resources 6.8 (Huang da et al., 2009a, b). Phosphopeptides with a fold change of at least 2 in duplicate samples after treatment with each drug were selected as a list of altered phosphopeptides. Phosphopeptides detected in duplicate samples were used as a background list. Enrichment *p* values of < 0.05 were considered significant.

**References**

Horn, H., Schoof, E.M., Kim, J., Robin, X., Miller, M.L., Diella, F., Palma, A., Cesareni, G., Jensen, L.J., Linding, R., 2014. KinomeXplorer: an integrated platform for kinome biology studies. Nature methods 11, 603-604.

Huang da, W., Sherman, B.T., Lempicki, R.A., 2009a. Bioinformatics enrichment tools: paths toward the comprehensive functional analysis of large gene lists. Nucleic Acids Res 37, 1-13.

Huang da, W., Sherman, B.T., Lempicki, R.A., 2009b. Systematic and integrative analysis of large gene lists using DAVID bioinformatics resources. Nat Protoc 4, 44-57.

Kitao, H., Morodomi, Y., Niimi, S., Kiniwa, M., Shigeno, K., Matsuoka, K., Kataoka, Y., Iimori, M., Tokunaga, E., Saeki, H., Oki, E., Maehara, Y., 2016. The antibodies against 5-bromo-2'-deoxyuridine specifically recognize trifluridine incorporated into DNA. Sci Rep 6, 25286.

Shannon, P., Markiel, A., Ozier, O., Baliga, N.S., Wang, J.T., Ramage, D., Amin, N., Schwikowski, B., Ideker, T., 2003. Cytoscape: a software environment for integrated models of biomolecular interaction networks. Genome Res 13, 2498-2504.
