# Supplementary material for: Panitumumab interaction with TAS‐102 leads to combinational anticancer effects via blocking of EGFR‐mediated tumor response to trifluridine
Source: Mol Oncol. 2017 May 30;11(8):1065–77. doi: 10.1002/1878-0261.12074 (PMC5537908; doi:10.1002/1878-0261.12074)
Supplement: Supplementary file 3 — Table S1. List of genes from the KEGG pathways identified by phosphoproteomic analysis, showing the effects of FTD versus control in LIM1215 cells. Table S2. List of genes from the KEGG pathways identified by phosphoproteomic analysis, showing the effects of panitumumab versus control in LIM1215 cells. [file MOL2-11-1065-s003.docx]

**Supplementary Table S1. List of genes from the KEGG pathways identified by phosphoproteomic analysis, showing the effects of FTD versus control in LIM1215 cells.**

| KEGG Pathway | *p* value | Gene Symbol | |
| --- | --- | --- | --- |
|  |  | Upregulated | Downregulated |
| Fanconi anemia pathway | 0.000 | *ATR ATRIP BLM BRCA1 BRCA2 BRIP1 FAN1 FANCI PALB2 POLH RPA2 USP1* | *FANCD2 FANCI RPA1* |
| RNA transport | 0.001 | *ACIN1 ALYREF EIF4G1 NCBP1 NUP153 NUP188 NUP214 NUP35 PNN POM121 POM121C POP1 POP4 RANBP2 RGPD3 RGPD4 RGPD8 RPP30 TPR XPO5* | *EIF3B NUP107 NUP133 NUP153 NUP210 NUP35 NUP50 PABPC1 PABPC1L PABPC3 PABPC4 POM121 POM121C RANBP2 RANGAP1 RGPD1 RGPD2 RGPD3 RGPD4 RGPD8 SRRM1 TPR XPO1* |
| Ribosome biogenesis in eukaryotes | 0.006 | *BMS1 MDN1 NOL6 NOP58 POP1 POP4 RBM28 RPP30 UTP14A* | *DKC1 GNL3 NOP56 TCOF1 WDR75 XPO1* |
| MicroRNAs in cancer | 0.014 | *BRCA1 CCNE1 CD44 CDCA5 EGFR ERBB2 MDM2 MET SHC1 SIRT1 SOS1 TP53* | *ABCC1 CDKN1B ERBB2 HNRNPK MAPK7 MARCKS MYC PAK4 PDCD4* |
| Cell cycle | 0.018 | *ATR BUB1B CCNE1 CDC27 CDK1 CHEK1 CHEK2 ESPL1 MCM3 MCM6 MDM2 PRKDC SMC1A SMC3 TP53* | *ANAPC2 BUB1 CDC20 CDKN1B GSK3B MCM6 MYC SMAD2 TTK* |
| Homologous recombination | 0.021 | *BLM BRCA2 NBN RAD50 RPA2* | *RPA1* |
| p53 signaling pathway | 0.022 | *ATR CCNE1 CDK1 CHEK1 CHEK2 DDB2 GTSE1 MDM2 STEAP3 TP53* | *GTSE1* |
| Focal adhesion | 0.047 | *ARHGAP35 ARHGAP5 BAD EGFR ELK1 ERBB2 FLNA JUN MET PXN SHC1 SOS1 TLN1 ZYX* | *ARHGAP5 ERBB2 FLNA FLNB GSK3B ITGB4 PAK1 PAK2 PAK4 ZYX* |

**Supplementary Table S2. List of genes from the KEGG pathways identified by phosphoproteomic analysis, showing the effects of panitumumab versus control in LIM1215 cells.**

| KEGG Pathway | *p* value | Gene Symbol | |
| --- | --- | --- | --- |
|  |  | Upregulated | Downregulated |
| Progesterone-mediated oocyte maturation | 0.000 |  | *ADCY3 BUB1 CDC25B HSP90AB1 MAPK1 MAPK3 RPS6KA1 RPS6KA3* |
| Estrogen signaling pathway | 0.002 | *CREB3L1* | *ADCY3 EGFR HSP90AB1 MAPK1 MAPK3 SHC1* |
| Oocyte meiosis | 0.026 |  | *ADCY3 BUB1 MAPK1 MAPK3 RPS6KA1 RPS6KA3* |
| Bladder cancer | 0.036 | *DAPK1* | *EGFR MAPK1 MAPK3* |
| Adrenergic signaling in cardiomyocytes | 0.046 | *CREB3L1* | *ADCY3 MAPK1 MAPK3 SLC9A1* |
